# BAFF, APRIL, TWEAK, BCMA, TACI and Fn14 Proteins Are Related to Human Glioma Tumor Grade: Immunohistochemistry and Public Microarray Data Meta-Analysis

**DOI:** 10.1371/journal.pone.0083250

**Published:** 2013-12-20

**Authors:** Vassiliki Pelekanou, George Notas, Marilena Kampa, Eleftheria Tsentelierou, Efstathios N. Stathopoulos, Andreas Tsapis, Elias Castanas

**Affiliations:** 1 Laboratories of Experimental Endocrinology, University of Crete, School of Medicine, Heraklion, Greece; 2 Laboratories of Pathology, University of Crete, School of Medicine, Heraklion, Greece; 3 INSERM U976, Hôpital Saint Louis, Paris, France; (4) Université Paris Diderot, Paris, France; University of Florida, United States of America

## Abstract

Gliomas are common and lethal tumors of the central nervous system (CNS). Genetic alterations, inflammatory and angiogenic processes have been identified throughout tumor progression; however, treatment still remains palliative for most cases. Biological research on parameters influencing cell survival, invasion and tumor heterogeneity identified several cytokines interfering in CNS inflammation, oxidative stress and malignant transformation, including TNF-superfamily (TNFSF) members. In this report we performed a meta-analysis of public gene-array data on the expression of a group of TNFSF ligands (BAFF, APRIL, TWEAK) and their receptors (BAFF-R, TACI, BCMA, Fn14) in gliomas. In addition, we investigated by immunohistochemistry (IHC) the tumor cells' expression of these ligands and receptors in a series of 56 gliomas of different grade. We show that in IHC, BAFF and APRIL as well as their cognate receptors (BCMA, TACI) and Fn14 expression correlate with tumor grade. This result was not evidenced in micro-arrays meta-analysis. Finally, we detected for the first time Fn14, BAFF, BCMA and TACI in glioma-related vascular endothelium. Our data, combined with our previous report in glioma cell lines, suggest a role for these receptors and ligands in glioma biology and advance these molecules as potential markers for the classification of these tumors to the proliferative, angiogenic or stem-like molecular subtype.

## Introduction

Gliomas are brain neoplasms that arise from glial cells and represent the most common primary tumors of the central nervous system (∼30%), with high lethality, approaching 80% in the first year of diagnosis. Despite their limited metastatic potential, gliomas are characterized by increased mortality, mainly attributed to high rates of local invasion and angiogenesis, accompanied by immunosuppression. Moreover, late diagnosis results in advanced stage/grade tumors and contributes to impaired prognosis, as high-grade gliomas are usually accompanied by broad vascular infiltration, with extensive hypoxic areas and necro-inflammatory features [1]. Gliomas are classified histologically as astrocytic, oligodendrocytic and oligoastrocytic tumors. In addition to this histological classification, a four-tiered grading system has been introduced in 2002 by the World Health Organization (WHO), proposing four grades of ascending malignancy: low grade (WHO grade I–II), anaplastic (grade III) and glioblastoma multiform (grade IV) [2]. The last WHO Classification of Tumors of the Central Nervous System was introduced in 2007, integrating morphological features, growth pattern and molecular profile of neoplastic cells [3]. It defined malignancy grade with an adequate prognostic relevance, providing important information for the clinical setting and the choice of therapeutic regimen [4]. Our knowledge on glioma behavior has been recently enriched by gene microarray analysis which has revealed specific subtypes, with distinct gene signatures, as well as predictive and prognostic relevance [5]–[8]. This transcriptome investigation revealed previously undescribed subclasses, with neural stem cell, proliferative, angiogenetic or mesenchymal traits, strengthening the hypothesis of multiple cellular origins in the genesis of gliomas and eventual transition from one type to another, throughout disease progression.

Inflammation can be a pluripotent promoter of tumor initiation, promotion and progression (see[9], for a review). During this process, an array of soluble mediators, produced by tumor cells or supplied by the tumor microenvironment/infiltrating cells, accounts for complex interactions influencing differentiation, activation, function and survival/apoptosis of multiple cell types. Among these mediators, members of the Tumor Necrosis Factor Superfamily (TNFSF, 19 ligands and 29 receptors) hold a prominent place, orchestrating a wide range of biological functions within the immune system and extra-immune cells [10]. A subset of this system of ligands and receptors has gained significant attention recently, being identified in different tumors (see below). It consists of the ligands APRIL (A Proliferation Inducing Ligand) and BAFF (B-cell Activating Factor of the TNF family) (TNFSF13 and 13B respectively) and their receptors BCMA (B-cell Maturation Antigen), TACI (Transmembrane Activator and CAML Interactor) and BAFFR (BAFF Receptor) (TNFR17, 13B and 13C). APRIL binds with high affinity to BCMA and TACI, while BAFF, sharing a lower affinity interaction with these two receptors, binds mainly to BAFFR (reviewed in [11]. Binding to their cognate receptors triggers diverse signaling pathways, involving, Nuclear Factor kappa-B (NFκB), and/or mitogen-activated kinases [12], [13]. These pathways have also been reported to participate in the evolution of glial cell tumors [14]. Another TNFSF member, the TNF-like WEAK inducer of apoptosis (TWEAK) and its receptor Fibroblast growth factor-inducible-14 (Fn14) (TNFSF12 and TNFRSF12A respectively), triggers tissue-specific pleiotropic effects and holds a distinct role in epithelial malignancies, [15]-[18]. BAFF, APRIL, and their receptors (BAFFR, BCMA and TACI) in addition to their well-established role in the B-lymphocyte network [19]–[22] have also been identified in an array of normal and neoplastic cell lines [14], [23]–[29] and tissues [30]–[36] of various origin. In previous reports, we identified an active role of these molecules in normal and pathological non-hemopoietic tissues [13], [30], [34], [37], as well as in pluripotent adult mesenchymal cells [30]. Through NFκB activation and newly identified signaling cascades [13], [29], these molecules are involved in cell cycle arrest and blockade of cell proliferation.

BAFF has been previously detected in normal brain and spinal fluid. In cultured human astrocytes, BAFF is secreted upon interferon- and TNF-stimulation [38]. This ligand is up-regulated in multiple sclerosis (MS) plaques, localized in astrocytes in the vicinity of BAFFR-expressing immune cells. Neither TACI nor BCMA transcripts have been identified so far in normal brain or MS plaques. Furthermore, both BAFF and BAFFR are expressed in neuronal cells and play a role in neuronal survival *in vitro* and in an *in vivo* murine model of inherited Amyotrophic Lateral Sclerosis [39]. APRIL has been identified in reactive but not in quiescent astrocytes and microglia and is also up-regulated within MS plaques [40]. Finally, TWEAK and its receptor Fn14 expression was identified mainly in astrocytes, but also in microglia, and neurons [41], displaying a variable expression in different normal brain areas. In addition, TWEAK and Fn14 have been found in neurons of the cerebral cortex, caudate nucleus, putamen, substantia nigra, cerebellar Purkinje cells and spinal cord and endothelial cells of medium- and small-caliber blood vessels. The striking abundant TWEAK/Fn14 immunostaining in perivascular structures, is indicative of their regulatory role in the neurovascular unit [42]. Based upon in vitro and in vivo animal studies, TWEAK has been reported to participate in multiple biological functions in the CNS, such as glial cell proliferation, demyelination, proinflammatory functions differentiation of adult neural progenitor cells, permeability of the blood-brain barrier and, apoptosis, under specific experimental conditions [43]–[51]. Moreover, TWEAK/Fn14 expression is enhanced in MS [44], [52]–[54] and stroke [55].

We have previously reported that APRIL promoted proliferation of glioblastoma-derived cell lines [29] and protected them from CD95- and TRAIL-mediated apoptosis [14], while TWEAK was found to protect glioma cell growth/progression (discussed in [56]. Through a positive feedback mechanism leading to elevated and sustained Fn14 expression, TWEAK and its cognate receptor are associated with gliomas' grade and inversely correlate with patients' survival [45], [57]. In view of the CNS pleiotropic effect of these molecules, in this study we have performed a systematic meta-analysis of public microarray data, seeking the expression of BAFF, APRIL and TWEAK and their cognate receptors (BAFFR, BCMA, TACI, Fn14) mRNAs. In addition, we investigated by immunohistochemistry the expression of BAFF, APRIL and TWEAK, as well as their receptors in a series of 56 gliomas, in order to identify possible new biological signatures in the complex behavior of these neoplasms.

## Materials and Methods

### Ethics Statement

This study was approved by the Research and Ethics committee of the University Hospital of Heraklion. Paraffin blocks of gliomas were retrieved retrospectively from the tissue archives of the Department of Pathology. As this was a retrospective study on surgical specimens, after the establishment of diagnosis and the application of appropriate treatment to patients and that this exploratory analysis would not derive on novel therapeutic modalities to involved patients, an individual informed consent was not considered necessary by the Ethics Committee.

### Analysis of public gene-array data

Studies were retrieved from Array Express Repository (http://www.ebi.ac.uk/arrayexpress/experiments) by using the keyword “glioma” and the following filters: Organism: Homo Sapiens, All arrays, All assays by molecule and Array assays. We retrieved 423 experiments. They were manually curated to include only microarray experiments in patients' samples. We have retained 39 studies, integrating 2083 samples (Table S1, Flowchart S1). Whenever a study re-analyzed previous ones it was the only one retained for analysis. In the repositories, meta-data were preprocessed and therefore additional preprocessing was not made.

As a first step, a correspondence with the GEO Database (http://www.ncbi.nlm.nih.gov/gds) was established (it retrieved 338 datasets). Thirty four studies were common in the two repositories, while 3 and 2 studies were unique in GEO and Array Express respectively. Values for the seven molecules of interest (APRIL/TNFSF13, BAFF/TNFSF13B, TWEAK/TNFSF12, BAFFR/TNFRSF13C, TACI/TNFRSF13B, BCMA/TNFRSF17, Fn14/TNFRSF12) were retrieved using the online tool GEO2R (http://www.ncbi.nlm.nih.gov/geo/geo2r) whenever possible (37 studies) and manually retrieved in two studies (Flowchart S1). Clinicopathological data (including histological types and/or WHO_Grade classification) were retrieved from sdrf files from the Array Express repository (Table S1, Checklist S1). Whenever WHO Grade was not included or was estimated by an earlier WHO classification, tumor samples were annotated according to the latest one (2007 WHO classification) [3].

If not already contained in metadata files, retrieved data were normalized by Log2-transformation before use. Some studies contain, in addition to disease states, control data, which were also used in the meta-analysis. In all other cases, a pooled mean and variance of non-tumoral values per technology were calculated, as previously described [58] and used in the analysis. In cases where metadata were reported as changes over control, a control mean of 0 or 1, as per study, was used with the same number of cases and variance/SD as for tumor samples. In all cases, mean value and SD were calculated and meta-analysis was performed by the use of the MetaXL program (EpiGear International Pty Ltd) in Microsoft's Excel, reporting Cohen's d and standardized mean differences, using random effects analysis [59]. Finally, standardized means were analyzed with ANOVA and correlation, with the SPSS V 21.0 program (IBM SPSS Statistics). For the retained studies, in which a significant difference was identified for each of the seven parameters, microarray data available through www.oncomine.org were further analyzed for a significant co-expression of these TNFSF members with other genes. Finally, lower grade gliomas (83 provisional samples) and glioblastomas (206 samples) data from the Cancer Genome Atlas ((http://cancergenome.nih.gov/) were queried, using the cBioPortal for Cancer Genomics (http://www.cbioportal.org/public-portal/) for estimating the impact of these gene alterations on patients' survival.

### Tumor specimens

Fifty six (56) glioma formalin-fixed paraffin-embedded specimens were retrieved from the Department of Pathology data base. Thirteen (13) specimens were low grade astrocytomas (three WHO grade I, including two pilocytic astrocytomas, one infantile ganglioglioma and ten grade II, including six astrocytomas and 4 oligodendrogliomas). Ten (10) cases of anaplastic gliomas (WHO grade III) and thirty three (33) cases of polymorphic astrocytoma-glioblastoma (WHO Grade IV) were also retrieved. Twelve serial sections (3 µm) were cut from each tissue-block. One was stained with hematoxylin and eosin and sequential slides were used for the immunohistochemical detection of APRIL BAFF, TWEAK, BCMA, TACI, BAFF-R and Fn14, plus a negative control. The slides were reviewed by two investigators independently and blindly (VP, ENS) and the final conclusion was reached in consensus. In few cases with discrepancy between the two observers, greater than 10%, the immunostained slides were reviewed in a double viewing microscope, so that the discrepancy was settled.

### Immunohistochemistry

After deparaffinization and hydration, slides were subjected to three cycles (5 min) of citrate buffer (0.01 M, pH 6.0) incubation in a microwave oven (500 W), and treated with 3% hydrogen peroxide for 15 min. They were incubated with primary antibodies, as previously described [34] and detailed in Table S2, together with the detection kits used. Counterstaining was performed using Harris hematoxylin. Known positive external controls, detailed previously [30], [37], as well as substitution of normal serum in place of the primary antibody were used in every run. An additional direct immunofluorescence assay was performed for BCMA detection in vascular endothelium, where DAPI was used for counterstaining. Additional immunostainings for CD31, CD68, CD3 and CD20 were used for further evaluation of endothelial and inflammatory cells respectively.

Slides were evaluated for the presence and the intensity of staining (expressed in a scale of 1–3 and the percentage of positive cells). The Histology score (H-score,[60]) was used for the analysis of results, calculating the intensity and the percentage of staining by the formula (%*1+%*2+%*3), ranging from 0–300. Cases with an H-score <25 were marked as negative.

### Statistical Analysis

Statistical analysis was performed by the use of appropriate parametric and non-parametric tests, as described in the Results section, by the use of SPSS v 21 (IBM, SPSS Inc, Chicago, IL). For multiple comparisons the Bonferroni adjustment was always applied. The statistical significance was set at 0.05.

## Results

### APRIL, BAFF and their receptor (BCMA, TACI, BAFFR) mRNA expression meta-analysis in gliomas

In meta-analysis, all five molecules expressed variable results in meta-analysis (Figures S1–S5). No distinct pattern, following tumor grade, was identified. However, although not significant, low grade tumors express a higher level of TNFSF molecule mRNA as compared to high grade ones. As results of meta-analysis were reported ad standardized mean differences, we have further performed an ANOVA analysis of these means, by WHO grade. No significant differences between WHO grade and either parameter were found. In correlation analysis, the only significant correlation spotted was that between APRIL and BAFF (Spearman's rho = 0.402, p<0.012) and BCMA and TACI (Spearman's rho = 0.303, p<0.015) (Table S3), indicating that, in gliomas, a combined transcription of ligands (BAFF, APRIL) or receptors (BCMA, TACI) occurs. We further report that APRIL was co-expressed with CXCL12, CCL11 and IL16 [61], IL2Rγ, CD4 and CD52 [62] and IL10R, TBXAS1 and CD86 [63], while BAFF was co-expressed with β-microglobulin and CX3CR1 [64], IgSF21 [65], CX3CR1 [62] or TNFSF10 (TRAIL) and the chemokines CXCL9, 10 and 11 [63], suggesting that these ligands are part of the immune response of glioma tumors. Finally neither the expression of the ligands (APRIL, BAFF) nor receptors (BAFFR, TACI, BCMA) exhibited significant correlation with patients' survival in the Cancer Genome Atlas cohort of lower grade gliomas or glioblastomas.

### Immunohistochemical detection of BAFF, APRIL and their receptors (BCMA, TACI, BAFFR) in clinical specimens

We performed immunohistochemical detection in 56 cases of glioma for APRIL, 50 cases for BAFF, 51 cases for BCMA, 56 cases for BAFFR and 42 cases for TACI, due to exhaustion of some tumor specimens (Table S4).

**Figure 1:**
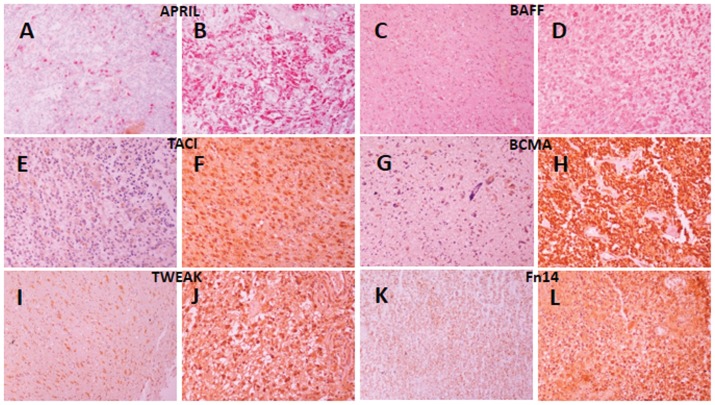
Representative immunohistochemical images of APRIL, BAFF, TWEAK and their cognate receptor expression in human gliomas. All pictures are taken under 20× magnification. APRIL immunoreactivity (A,B) increases from low grade to higher grade lesions. Note the intense staining in larger cells, while smaller ones are negative (A). BAFF immunoreactivity declines from lower grade (C, diffuse and intense), to higher grade gliomas (D). TACI displayed higher immunoreactivity in higher grade gliomas (F) than in low grade ones (E). Similar findings were found for BCMA as well (low grade G, high grade F). Low and high grade tumors displayed heterogeneous regarding percentage of positive cells and/or intensity of staining for TWEAK and Fn14. Here representative photos from Grade III gliomas for TWEAK (I, J) and Fn14 (K, L), respectively are shown.

APRIL stained all 56 tumors; immunoreactivity was always intracellular, restricted in granules, as previously described in human brain [40] (Figure 1A–B). In rare cases, in which normal appearing neurons were recognized, they were also positive for APRIL. Vascular endothelial cells were constantly negative (not shown).

**Figure 2:**
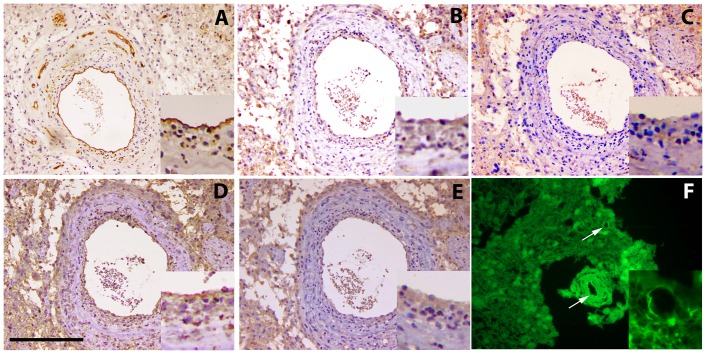
Representative images of BAFF, TWEAK and their cognate receptor BCMA, TACI and Fn14 expression in vascular endothelial cells of human glioma specimens. **Upper row:** vascular endothelium immunoreactivity for CD31 (**A**), Fn14 (**B**), and TWEAK (**C**). **Lower row**: vascular endothelium immunoreactivity for BAFF (**D**) and TACI (**E**). In panel **F**, vascular endothelial immunoreactivity for BCMA (direct immunofluorescence assay, with anti-BCMA-FITC antibody. Similar results were obtained by indirect immunostaining with non-fluorescent anti-BCMA antibody (not shown). Arrows show tumor vessels. APRIL was not detected in endothelial cells (not shown). All pictures are taken under 20× magnification. Bar = 200 µm. Inserts in each panel show a higher magnification of a representative part of the vessel.

A homogeneous BAFF immunoreactivity was observed in all tumors, with a higher intensity in low grade ones (Fig. 1C, D). Normal-appearing neurons were also positive for BAFF, in accordance with a recent report [39]. Vascular endothelial cells, positive for CD31 immunostaining (Figure 2A), were also found positive for BAFF (Figure 2B). Similar to APRIL, BAFF staining pattern was always intracellular, in some cases delimited in granular structures.

BAFFR staining was constantly negative in all glioma specimens examined (not shown). In contrast, TACI and BCMA were detected in all assayed specimens, with an increased intensity in tumors of higher grade (Figure 1E, F and G, H respectively). The expression pattern was cytoplasmic, mainly granular. Vascular endothelial cells were positive for both TACI and BCMA (Figure 2E and F respectively, arrows). Endothelial immunostaining for TACI and BCMA was confirmed by immunostaining with a CD31 specific antibody. The presence of BCMA in endothelial cells has also been verified by immunofluorescence, using both BCMA and CD31 specific antibodies (Figure 2). We have to note that the BCMA-specific antibody stains also normal brain neuronal cells and their axons (not shown).

**Figure 3:**
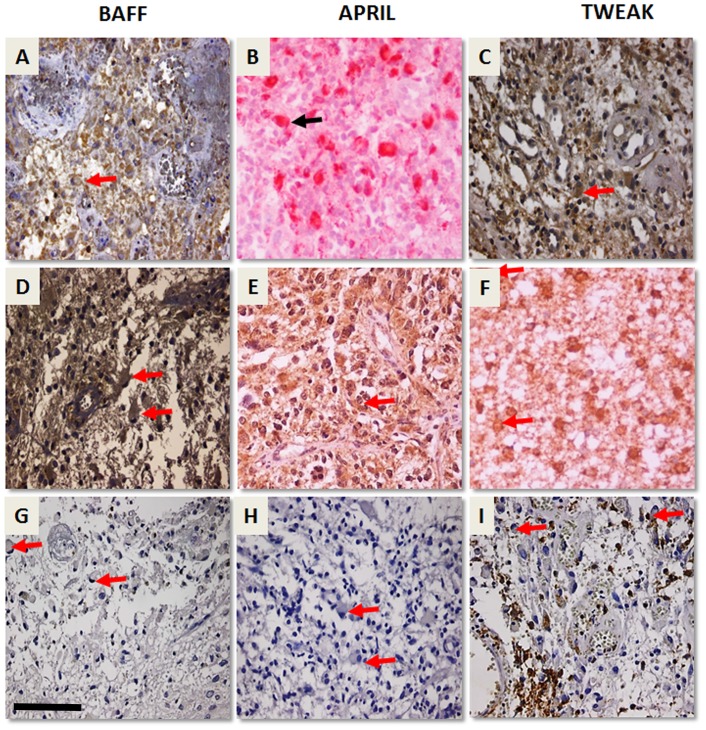
Representative images of BAFF, APRIL, TWEAK and their cognate receptors as well as inflammatory markers CD3, CD20, CD68 expression in human glioma specimens. Parallel immunostaining of BAFF, APRIL, TWEAK, BCMA, TACI, Fn14 and inflammatory markers CD3, CD20 and CD68 showing that the main source of these TNFSF members are reactive and tumoral glial cells *per se* in areas with minimal inflammation. Red arrows (black in 3B) indicate gemistocytes immunopositive for BAFF (**A**), APRIL (**B**), TWEAK (**C**), TACI (**D**), BCMA (**E**) and Fn14 (**F**). Gemistocytes negative for lymphocytic CD3 (**G**), CD20 (**H**) and panmacrophage CD68 (**I**) immunostaining. CD68 immunopositive macrophages/microglia and monocytes in I. All pictures are taken under 40× magnification.

To further confirm the above mentioned expression profile we enriched our immunohistochemical analysis with CD68, CD3 and CD20 immunostaining (Figure 3 and Figure S6), for microglia/macrophages and lymphocytic population, respectively. The pan-macrophage marker CD68 was mainly observed in microglia/macrophages (Figure 3I and Figure S6A), as well as intravessel monocytes. Microglia were scattered or clustered in perivascular areas, within large vessel wall or in close vicinity of neuronal cell bodies. In glioblastomas this immunopositivity was enhanced, especially in perinecrotic areas (although frankly necrotic areas were systematically avoided), with increased presence of CD68 immunopositive foamy macrophages and large granular cells, indicative of a degenerative nature. We underline the observation that reactive and neoplastic astrocytes, well represented by gemistocytes, were constantly negative for CD68 immunostaining (Figure 3I), whilst they were immunopositive for all of the TNFSF members we have examined (BAFF, APRIL, TWEAK, TACI, BCMA, Fn14) (Figure 3A–F) The lymphocytic immunostaining on the other hand (Figure 3G, H and Figure S6B, C), was less evident, with CD20 immunopositive lymphocytes being scant. Lymphocytes were present in the lumen of blood vessels, or clustered perivascularly forming lymphocytic cuffs. Immunostaining for BAFF, APRIL, TACI, BAFFR, TWEAK and Fn14 was positive in lymphocytes (Figure S6D–G). No gemistocyte was evidenced immunopositive for CD3 or CD20 (Figure 3G, H, respectively).

**Figure 4:**
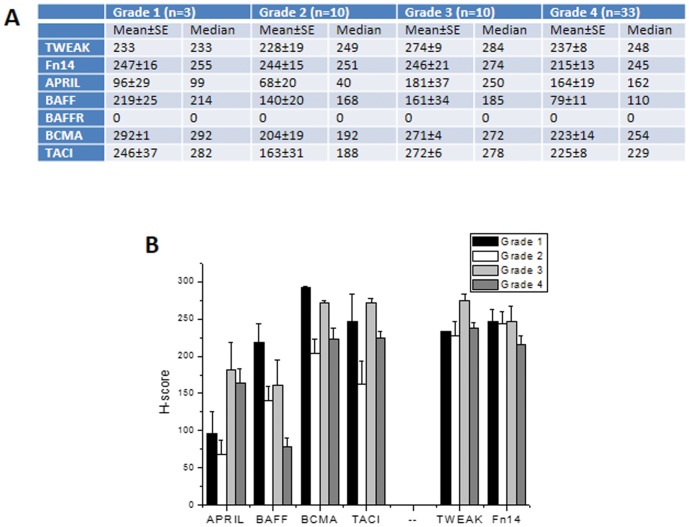
Quantification of BAFF, APRIL, TWEAK and their receptors in gliomas, stratified by grade. Data are expressed as mean±SEM and medians of the corresponding H-score of tumors, presented in Table S4 in **A** and bar graph (mean±SEM) in **B**.

Quantification of the immunohistochemical staining (Table S4, Figure 4) revealed that APRIL presents an increased staining intensity (as expressed by H-score) with tumor grade, while BAFF staining intensity decreases in tumors of higher grade. This was further confirmed by correlation analysis, using the distribution-free Spearman's correlation coefficient (p = 0.041 and 0.016 for APRIL and BAFF respectively). When tumors were grouped in low (WHO I–II) and high grade (WHO III–IV) a significant increase of APRIL, BCMA and TACI expression was detected in the high grade group (Table 1), as compared to that of low grade group.

**Table 1:** Quantification of BAFF, APRIL, TWEAK and their receptors in Low (WHO I & II) and high grade gliomas (WHO grade III & IV).

	Low Grade (n = 13)		High Grade (n = 43)		Significance (ANOVA)
	Mean±SE	Median	Mean±SE	Median	one way
TWEAK	229±15	247	246±7	254	p = 0.077
Fn14	246±12	270	223±11	245	p = 0.404
APRIL	77±17	41	168±16	163	p = 0.050
BAFF	144±17	176	98±12	115	p = 0.291
BAFFR	0	0	0	0	
BCMA	214±17	192	235±11	263	p = 0.033
TACI	177±28	190	237±7	242	p = 0.030

Data are expressed as mean±SEM and medians of the corresponding H-score of tumors, presented in Table S4. Statistical analysis was performed by one way ANOVA, with Bonferroni correction.

In conclusion, our histological data identified an enhanced APRIL, TACI and BCMA protein expression in high grade gliomas. Contrariwise, BAFF protein expression has been found decreased in high-grade as compared to that of low-grade tumors. Finally, vascular endothelial cells, exhibited immunoreactivity for BAFF, BCMA and TACI molecules.

### Transcriptional signature of TWEAK-Fn14 in microarray data

Meta-analysis of public transcriptomic studies for TWEAK and Fn14 in gliomas did not reveal any statistical difference among tumors of different grades (Figures S7 and S8 respectively). Here too, results were highly heterogeneous. ANOVA analysis of mean standardized differences by tumor grade did not attain statistical significance (p<0.15), nor did post-hoc group comparison after Bonferroni adjustment. Correlation analysis did not show any significant correlation between this ligand/receptor couple. However, significant correlations were found between APRIL and both TWEAK and Fn14 (Spearman's rho = 0.576, p<0.001 and 0.333, p<0.01 respectively), as well as between BCMA and Fn14 (rho = 0.268, p<0.026), suggesting a common regulation of these genes, or due to their co-expression in vascular endothelium (see below). Fn14 was found to be co-expressed with IGFBP2 and 3 and VEGFA [62], IFNγR2 [64], TNFR1A and IGFBP2 [66] and lamin A [63], [65], suggesting a possible role of this receptor in glioblastoma angiogenesis.

Mining the Cancer Genome Atlas cohort, in lower grade gliomas, TWEAK/Fn14 modified expression (7/83 cases) was associated with lower survival (10.35/75.10 months, p<10^−4^); in contrast, in glioblastoma cases (585 tumors), in which only 1% presented an altered expression of either TWEAK or Fn14, no significant correlation with patients' overall survival (16.57 vs 13.57 months, p<0.553) was evidenced.

### Immunohistochemical detection of TWEAK and Fn14

TWEAK immunoreactivity was studied in 36 gliomas, for which sufficient tissue was available, while Fn14 was assayed in 54 specimens. Immunoreactivity of both TWEAK and Fn14 was heterogeneous; normal-appearing brain tissue was constantly negative (not shown); low and high-grade tumors, on the other hand, presented a heterogeneous staining (Figure 1I, J and K, L, respectively). Vascular endothelium was positively stained for both TWEAK and Fn14 (Figure 2B and C, respectively, arrows), a result confirmed by CD31 immunostaining. As shown in Figure 3, median TWEAK immunoreactivity was constant in grade I, II and IV tumors, while it increased significantly only in grade III. Contrariwise, mean Fn14 immunoreactivity was constant in grade I–III tumors and significantly decreased in grade IV.

We have further explored whether TWEAK/Fn14 are co-expressed with the other ligand/receptors of the TNFSF members, we have assayed. As shown in Table 2, we found a negative correlation between APRIL and Fn14 protein (p<0.001). In addition we report a significant positive correlation between BAFF and TACI (p<0.023).

**Table 2:** Correlation between pairs of the TNFSF ligands and receptors identified in our series of 56 gliomas.

		TWEAK	Fn14	APRIL	BAFF	BCMA	TACI
TWEAK	Rho	1	0.197	0.125	0.159	0.123	0.289
	Sig.	.	0.125	0.233	0.177	0.241	0.061
	N	36	36	36	36	35	30
Fn14	Rho		1	−0.410**	−0.2	0.138	0.02
	Sig.		.	0.001	0.07	0.162	0.45
	N		56	56	56	53	42
APRIL	Rho			1	0.155	0.142	0.242
	Sig.			.	0.127	0.156	0.061
	N			56	56	53	42
BAFF	Rho				1	0.039	0.310*
	Sig.				.	0.39	0.023
	N				56	53	42
BCMA	Rho					1	0.156
	Sig.					.	0.162
	N					53	42
TACI	Rho						1
	Sig.						.
	N						42

The Spearman's correlation coefficient (rho) is reported, together with the number of cases and statistical significance. H-score (as presented in Table S4 is used for the analysis.

*. Correlation is significant at the 0.05 level (1-tailed).

**. Correlation is significant at the 0.01 level (1-tailed).

In conclusion, our data reveal a differential expression of the studied TNFSF members and their receptors in gliomas. The protein expression is not limited to inflammatory infiltrate, but is mainly due to the specific expression of these molecules by glioma cells *per se* and distinct structures, like the vascular endothelium. However, a distinct pattern of transcription of these molecules in gliomas is not evidenced in micro-array data, possibly due to the heterogeneity of the tissue samples used and the inavailability of exclusive glioma cells nucleic acid extracts in the majority of the studies we reanalyzed.

## Discussion

The spectrum of cytokines involvement in CNS homeostasis or disease remains unclear. Among them, TNFα is locally synthesized in the CNS with several important functions, including injury-mediated microglial and astrocyte activation, regulation of blood-brain barrier permeability, stimulation of the recruitment of neutrophils and monocytes, febrile responses, glutamate transmission and synaptic plasticity [67], [68], while anti-TNFα antibodies have been used in brain pathologies, with impressive results [68], [69]. Other TNF superfamily (TNFSF) members have been tried as therapeutic agents in gliomas [40], [70]–[72], with TRAIL being the best-studied [73]–[78].

In the present study we have assayed for the first time by immunohistochemistry 56 gliomas of various grade for the expression of a subset of TNFSF members, namely APRIL, BAFF and TWEAK, as well as their cognate receptors (BCMA, TACI, BAFF-R and Fn14), in parallel with a systematic search and meta-analysis of available public gene-array data for possible over- or under-expression of these molecules in gliomas, as compared to normal brain. We report that all three ligands (APRIL, BAFF and TWEAK) are expressed in human glioma specimens by immunohistochemistry, in accordance with previous reports in glioma-deriving cell lines [29], [45], [46]. Our work presents important clues on the relation of these molecules with glioma grade, based on our immunohistochemical data. In contrast, the microarray meta- analysis hasn't revealed significant correlation of tumor grade with a specific transcription pattern, or patients' survival. Our data clearly indicate that a microarray approach of glioma tumors failed as far as it concerns the expression of the TNFSF members studied here, possibly due to the increased heterogeneity of the brain tissue used. Further work is needed to investigate whether this phenomenon is a general one or it concerns only a limited number of genes. In addition, in our immunohistochemical approach, we did not observe a significant correlation of these TNSFSF molecules with histologic subtypes, probably due to our limited number of samples.

APRIL and TWEAK immunoreactivity increased with tumor grade, while BAFF staining intensity was decreased in high grade gliomas, as compared to that of low grade. This result was not reflected in mRNA meta-analysis of more than 2000 cases. Notably, APRIL is reported quasi-absent in quiescent astrocytes and an enhanced production is described in activated astrocytes in cases or MS or other brain pathologies [40]. TWEAK, is present in all kinds of glial cells, and staining intensity follows tumor grade. However, this immunostaining is quite heterogeneous, in accordance with previous data suggesting that TWEAK/Fn14 expression in normal brain displayed a varying intensity, depending on brain topography [41]. This tandem of ligand-receptor further showed an increased expression in MS astrocytes, following the severity and time course of the disease [44], [52]–[54]. Concerning BAFF, we report a great discrepancy between glioma gene microarrays and immunohistochemical detection, as we show a decrease of immunoreactive BAFF expression, following tumor grade. The limited number of samples did not permit us to relate BAFF expression to astrocytomas, as astrocytes are considered the primary source of the molecule [38]. In contrast to previous microarray studies, in which BAFFR mRNA was present, but not modified in normal brain and glioma tissue, we did not evidence BAFFR immunopositive tumoral cells in our specimens. We should further underline that BAFFR was always absent in all kinds of tumors (epithelial or mesenchymal) we have studied so far [13], [30], [31], [34], [37], in spite of its detection at the mRNA level, suggesting a possible limited role of this receptor in solid tumor biology. Finally, we have detected a heterogeneous Fn14 immunostaining with staining intensity (H-score) quite stable within Grade I–III tumors and a decrease of immunostaining in glioblastomas. Strikingly, in a previous study that has stratified glioblastomas according to their transcriptional signature, Fn14 is suggested as a gene classifier between oligodendroglioma/astrocytoma-rich (WHO Grade II/III-rich) and glioblastoma-rich (WHO Grade IV-rich) subtypes [7]. It would be worthwhile to comment that comparison of the two main types, shows that the oligodendroglioma/astrocytoma-rich main type exhibited enhanced activities of exogenous hormone-stimulated growth and PAR1 signaling activity, while up-regulated gene profiles associated with the glioblastoma-rich main type, involved cell cycle/mitotic pathways, hypoxia, tumor necrosis, and NF-κB pathway signaling, consistent with a more malignant and aggressive tumor type.

Gliomas are highly vascular tumors, particularly prone to inflammatory infiltrate, which is a major source of the studied TNFSF molecules so far. However, in our immunohistochemical study, realized under the scope of verification of the cellular origin of these molecules in gliomas, we have identified these molecules in discrete tumor areas, devoted from apparent infiltrating immune cells, Indeed, they were detected in glial tumoral cells *per se*, as well as non tumoral adjacent CNS components (where present), like neurons. Additional CD68, CD3 and CD20 immunostaining for microglial/macrophages and lymphocytic population respectively, confirmed our observation on expression of the studied TNFSF molecules by glial cells *per se* (tumoral and reactive) (Figures 3 and 4). The lymphocytic infiltrate was minimal, with CD20 immunopositive lymphocytes being scant and CD3 immunopositive population being the most represented. Immunostaining for BAFF, APRIL, TACI, BCMA, BAFFR, TWEAK and Fn14 was positive in lymphocytes, as expected. On the other hand no gemistocyte was evidenced immunopositive for CD3 or CD20. In low grade gliomas the pan-macrophage marker CD68 was limited in scattered or perivascular and adjacent to neurons microglia/macrophages, as well as intravessel monocytes. This immunopositivity was enhanced in glioblastomas, especially in perinecrotic areas. Foamy macrophages and large granular cells (indicative of a degenerative nature) were the main source of CD68 immunostaining. Gemistocytic astrocytes, (Figure 3I), were constantly negative for CD68 immunostaining, whilst they were immunopositive for all of the TNFSF members we have examined (BAFF, APRIL, TWEAK, TACI, BCMA, Fn14) (Figure 3A-F). The omnipresence of BAFF, APRIL, TWEAK and receptors in gemistocytes merits further investigation. Indeed, while immunohistochemical investigations have emphasized the low proliferation index of the neoplastic gemistocytes, there is a striking tendency of gemistocytic astrocytoma WHO grade II or III lesions to rapidly progress to glioblastoma [79]. On the other hand, microglia exhibit different proliferative activities at different grades of malignancy, with the highest rates of proliferating microglia shown in pilocytic astrocytomas. The microglial accumulation in diffuse glial tumors is not limited to a nonspecific reaction to tissue injury but indicates active participation of these cells in promotion of astrocytoma invasive phenotype. Notably, tumor cells can occasionally be reactive to some macrophage markers, like CD68 (27, 34). Recently, glioma cells CD68 expression has been assigned of prognostic value, especially for astrocytomas [80]. However, large and more coarsely granular, glial tumor cells also resemble macrophages. Given their lysosomal content, the tumor cells may exhibit immunoreactivity for macrophage markers such as CD68, as observed in the present study as well. Moreover, occasional cells may have peripheral immunopositivity for GFAP, but most cells are negative. On the basis of both these findings it is most likely that the granular cells represent glioma cells with a distinct degenerative pathway [3].

Surprisingly, we identified three receptors (BCMA, TACI and Fn14), as well as their ligands BAFF and TWEAK, in gliomas' vascular endothelium. Previous studies demonstrated the expression of TWEAK/Fn14 in vascular structures and gland tubules [30], [34], [37], [81], positioning TWEAK as a major player in angiogenesis [82], [83]. BAFF, TACI, TWEAK and Fn14 have also been found expressed within adipose tissue endothelial cells, while APRIL and BCMA were absent [30].

We should underline the discrepancies among different transcriptomic reports, as well as the differences between microarray and immunohistochemical data. In microarray studies, this controversy might be attributed to high pleiomorphism and variability of cellular components, which, especially in glioblastomas, display a notoriously heterogeneous phenotypic presentation. Moreover, if gene-array experimental design does not target exclusively tumoral cells (i.e. using laser capture microdissection), it is difficult to decipher whether the cells of origin are tumoral, inflammatory or other. An additional layer of complexity has been added as transcriptional differences exist between cells of tumor core and invasive cells located in the peritumoral brain parenchyma [84]. Finally, some of the above TNFSF molecules exhibit differential expression within brain loci, so it is plausible to hypothesize that transcriptional signature could interfere with the topography of tissue sampling. An additional point of discrepancy is observed when cell lines and human tissues are compared, especially in view of receptors' expression (BCMA, BAFF-R, TACI, Fn14). Indeed, in our initial report [29], only APRIL, BCMA and TACI mRNAs were found in glioma cell lines, while BAFF mRNA is also expressed in tumor tissues, as detected in microarray studies. We should underline that this is not the first time we report discrepancies in expression of these molecules, between cell lines, primary cell cultures and tissue specimens [30].

In view of our data, which might be the role of these molecules in glioma biology? Although no mechanistic study was performed here, our previous work reports the presence of APRIL, but not BAFF in eight glioblastoma cell lines [29]. Normal embryonic astrocytes also express APRIL but not BAFF [29], in contrast to mesenchymal stem cells [30]. Recombinant BAFF promoted differentiation of adipose tissue-derived mesenchymal cells, whilst APRIL and TWEAK decreased it [30]. As these cells could also differentiate to neuronal nestin-rich precursors [85], it should be interesting to investigate the autocrine/paracrine role of these TNFSF members in the neuronal differentiation of mesenchymal cells. On the other hand, APRIL induced proliferation in 4/8 glioblastoma cell lines, mediated mainly through the BCMA receptor, while BAFF had no effect in any cell line tested. Interestingly, no BAFF binding could be detected in neuronal precursor cells, astrocyte progenitors or normal adult astrocytes [29]. In our tumor series, BAFF was detected in low-grade tumors preferentially, while a correlation with TACI was evidenced, evocative of a preferential interaction of this ligand/receptor couple, as proposed in previously published reports [86], [87]. Another element we report here is the co-expression of APRIL and BAFF mRNA with a number of cytokines and chemokine molecules, at the mRNA level, suggestive of a possible inflammatory swift of the tumor cells *per se*. Finally, TNFSF expressing glial cells *per se* could also affect the vascular density of gliomas as BAFF, TWEAK, BCMA, TACI and Fn14 expression was increased in the vascular endothelium (especially in small caliber vessels compared to characteristic hyalinized glioblastoma ones). This finding could provide hints on the eventual pro-angiogenic role of this set of ligands/receptors in gliomas. Indeed, rich vascularization is a common element of glioblastomas, correlating with their biological aggressiveness, degree of malignancy and recurrence rate [88] and a specific molecular subtype with markers of angiogenesis and mesenchymal features has been reported [5]. The co-expression of Fn14 with VEGFA [62], and lamin A found in microarray studies [63], [65] and with APRIL and BCMA, reported here, provides a mechanistic support for this possible pro-angiogenic action. Moreover, recent reports suggest the presence of TACI, BAFFR and BCMA within bone marrow endothelial cells. Secreted BAFF and APRIL by endothelial cells *per se* is presented as regulator of bone marrow endothelial niches on leukemia cell survival establishing bidirectional cross-talk between the stroma and B cells [89], [90].

Currently treatment decision-making is based mainly on histologic classification of gliomas and clinical parameters. However, differences between histologic subclasses and grades may be subtle, and glioma classification can be subject to a large inter-observer variability. This variability and undeniable complexity of gliomas have inspired a series of elegant molecular studies that reveal specific molecular subtypes of gliomas, with important prognostic relevance, delineation of patterns of disease progression and specific genes abnormalities [5]–[8]. Even if the different investigation groups have identified various gene signatures and deriving glioma subtypes, there is a common suggestion for mesenchymal traits of the most aggressive glioblastomas [5], [7], [8]. In addition to the progress in molecular genetics and pathogenesis of gliomas [91], recognition of the cell type(s) of origin remains a matter of debate. Until recently, high grade gliomas were presumed to arise from glial cells residing within the brain parenchyma. However, recent evidence in human and animal studies suggests neural stem cells as an alternate cellular origin of gliomas [92], [93]. Previous investigation of ours indicate that both ligands and receptors of the TNFSF may also be synthesized by solid tumors [30], [31], [34], [37]. Despite discrepancies, there are insights that in gliomas BAFF, APRIL, TWEAK and their receptors follow a distinct expression pattern, not identical to that observed in normal brain, neurodegenerative disease, other brain tumors, or extra CNS tissues; these molecules could provide hints for the classification of these tumors to the proliferative, angiogenic or stem-like molecular subtype. Finally, the expression of three receptors (BCMA, TACI and Fn14), as well as their ligands BAFF and TWEAK, in brain vascular endothelium suggests that this *ensemble* of ligands and receptors could play a role in glioma hypervascularization.

## Supporting Information

Figure S1 **Meta-analysis of human gliomas' micro-arrays.** Forest plot of APRIL expression in 2083 tumor glioma specimens, stratified according to their WHO grade. Results are presented as standardized mean differences (Cohen's d) between tumor and non-tumor samples.
(TIF)

Figure S2 **Meta-analysis of human gliomas' micro-arrays.** Forest plot of BAFF expression in 2083 tumor glioma specimens, stratified according to their WHO grade. Results are presented as standardized mean differences (Cohen's d) between tumor and non-tumor samples.
(TIF)

Figure S3 **Meta-analysis of human gliomas' micro-arrays.** Forest plot of BAFFR expression in 2083 tumor glioma specimens, stratified according to their WHO grade. Results are presented as standardized mean differences (Cohen's d) between tumor and non-tumor samples.
(TIF)

Figure S4 **Meta-analysis of human gliomas' micro-arrays.** Forest plot of TACI expression in 2083 tumor glioma specimens, stratified according to their WHO grade. Results are presented as standardized mean differences (Cohen's d) between tumor and non-tumor samples.
(TIF)

Figure S5 **Meta-analysis of human gliomas' micro-arrays.** Forest plot of BCMA expression in 2083 tumor glioma specimens, stratified according to their WHO grade. Results are presented as standardized mean differences (Cohen's d) between tumor and non-tumor samples.
(TIF)

Figure S6 **Representative images of inflammatory infiltrates in human glioma specimens.** CD68 immunopositive macrophages/microglia in perivascular area and within lymphocytic cuff (A). Lymphocytic infiltrate was minimal, mainly organized in lymphocytic cuffs. CD3 positive lymphocytes (B), and scant CD20 positive lymphocytes (C). Positive immunostaining for TNFSF molecules TACI (D), TWEAK (E), Fn14 (F) and BAFF (G) was oserved in glial tumoral cells, vascular endothelium and inflammatory infiltrate. All pictures are taken under 40× magnification.
(TIF)

Figure S7 **Meta-analysis of human gliomas' micro-arrays.** Forest plot of TWEAK expression in 2083 tumor glioma specimens, stratified according to their WHO grade Results are presented as standardized mean differences (Cohen's d) between tumor and non-tumor samples.
(TIF)

Figure S8 **Meta-analysis of human gliomas' micro-arrays.** Forest plot of Fn14 expression in 2083 tumor glioma specimens, stratified according to their WHO grade. Results are presented as standardized mean differences (Cohen's d) between tumor and non-tumor samples.
(TIF)

Table S1 **Identifiers of glioma micro-array studies used in the present work for the meta-analysis of the TNFSF members, including Supplementary References to these studies.**
(DOCX)

Table S2 **Primary antibodies and detection kits used in the study.**
(DOCX)

Table S3 **Non-parametric correlation of standardized mean differences of micro-array studies.**
(DOCX)

Table S4 **H-score of the TNFSF members in gliomas.**
(DOCX)

Checklist S1 **PRISMA checklist of the meta-analysis elements.**
(DOC)

Flowchart S1 **PRISMA Flowchart explaining the selection of studies included in the meta-analysis presented here.**
(DOC)
