# Supplementary figures and images for: BAFF, APRIL, TWEAK, BCMA, TACI and Fn14 Proteins Are Related to Human Glioma Tumor Grade: Immunohistochemistry and Public Microarray Data Meta-Analysis
Source: PLoS One. 2013 Dec 20;8(12):e83250. doi: 10.1371/journal.pone.0083250 (PMC3869762; doi:10.1371/journal.pone.0083250)

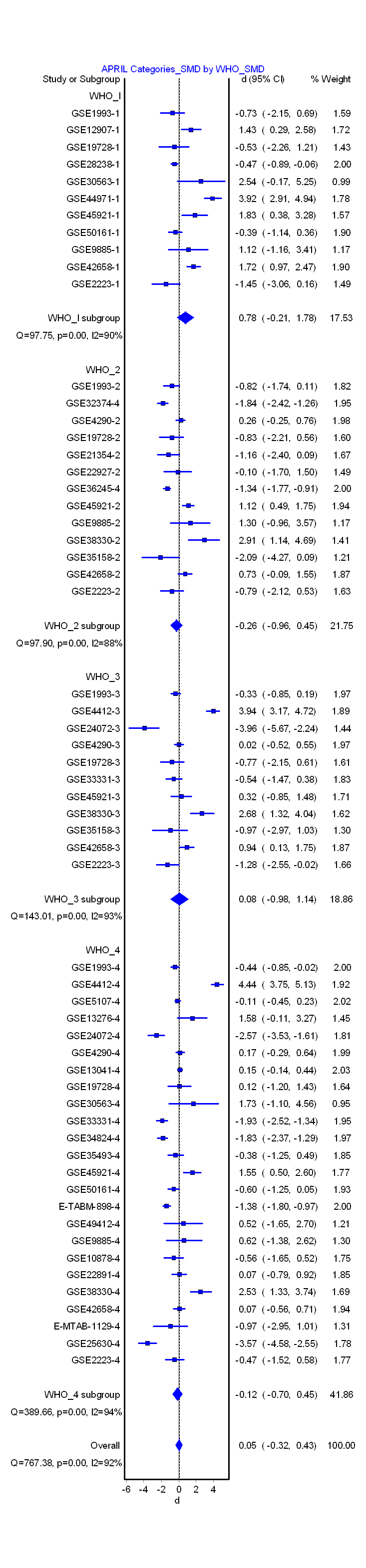

Supplement: Figure S1 — Meta-analysis of human gliomas' micro-arrays. Forest plot of APRIL expression in 2083 tumor glioma specimens, stratified according to their WHO grade. Results are presented as standardized mean differences (Cohen's d) between tumor and non-tumor samples. (TIF) [file pone.0083250.s001.tif]

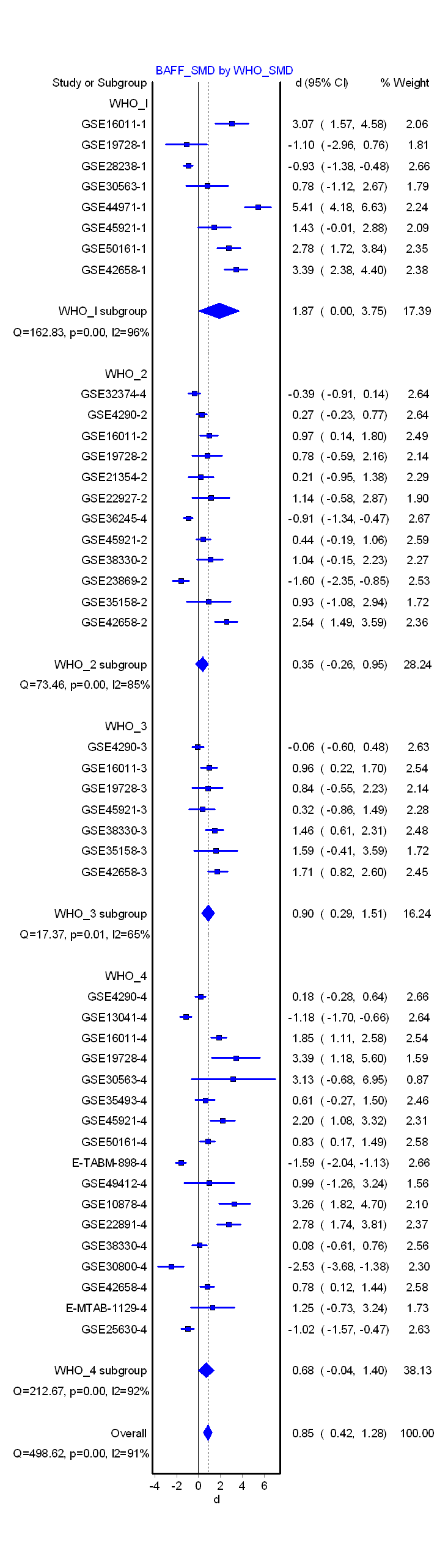

Supplement: Figure S2 — Meta-analysis of human gliomas' micro-arrays. Forest plot of BAFF expression in 2083 tumor glioma specimens, stratified according to their WHO grade. Results are presented as standardized mean differences (Cohen's d) between tumor and non-tumor samples. (TIF) [file pone.0083250.s002.tif]

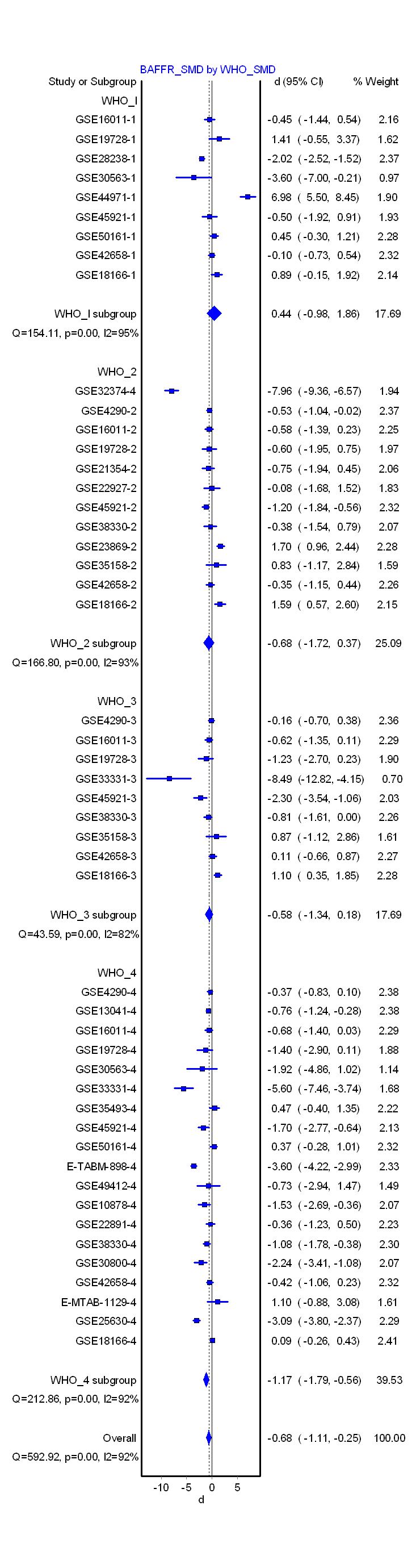

Supplement: Figure S3 — Meta-analysis of human gliomas' micro-arrays. Forest plot of BAFFR expression in 2083 tumor glioma specimens, stratified according to their WHO grade. Results are presented as standardized mean differences (Cohen's d) between tumor and non-tumor samples. (TIF) [file pone.0083250.s003.tif]

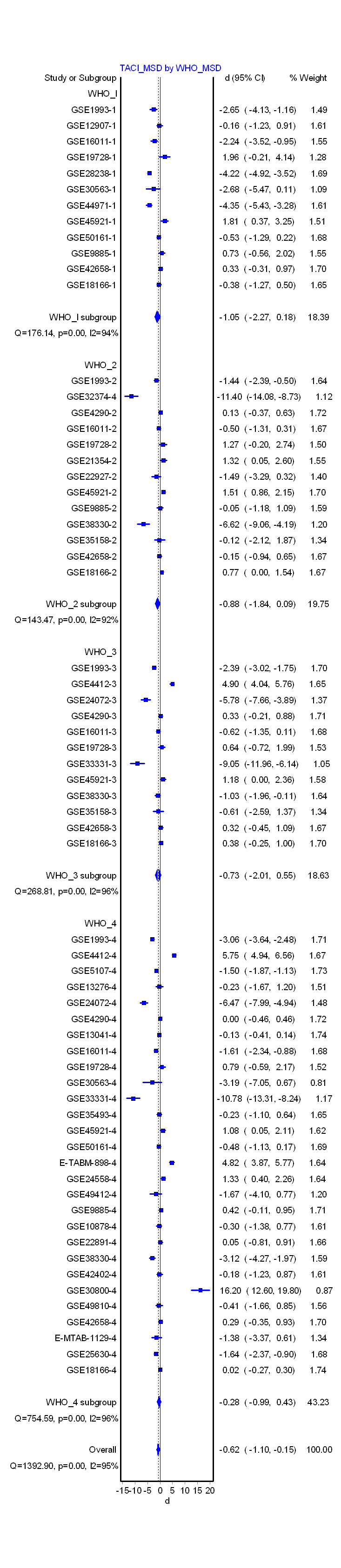

Supplement: Figure S4 — Meta-analysis of human gliomas' micro-arrays. Forest plot of TACI expression in 2083 tumor glioma specimens, stratified according to their WHO grade. Results are presented as standardized mean differences (Cohen's d) between tumor and non-tumor samples. (TIF) [file pone.0083250.s004.tif]

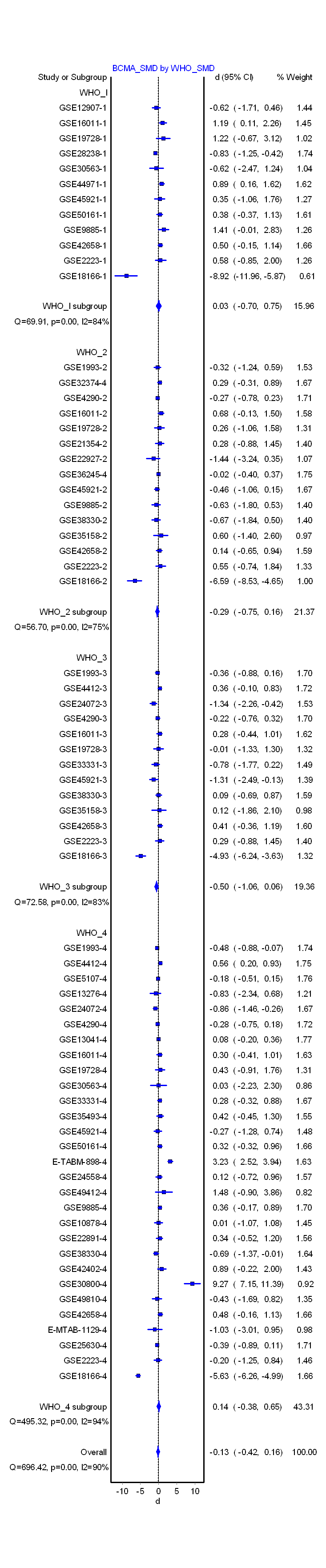

Supplement: Figure S5 — Meta-analysis of human gliomas' micro-arrays. Forest plot of BCMA expression in 2083 tumor glioma specimens, stratified according to their WHO grade. Results are presented as standardized mean differences (Cohen's d) between tumor and non-tumor samples. (TIF) [file pone.0083250.s005.tif]

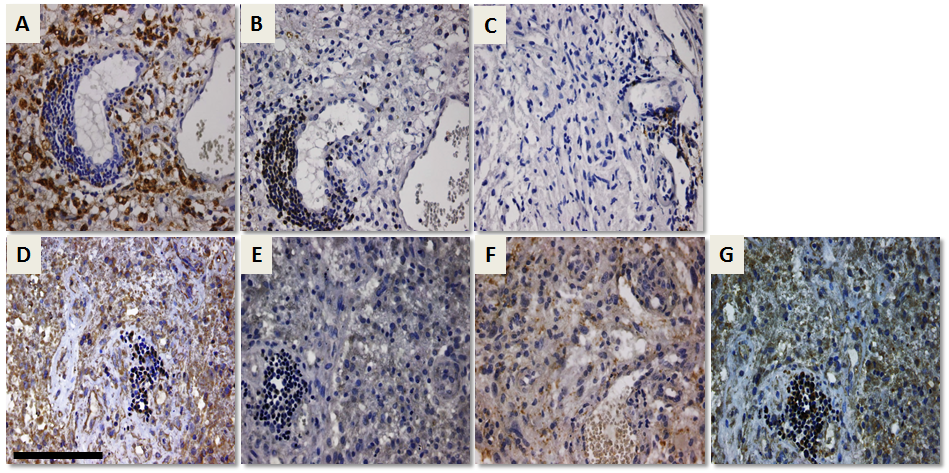

Supplement: Figure S6 — Representative images of inflammatory infiltrates in human glioma specimens. CD68 immunopositive macrophages/microglia in perivascular area and within lymphocytic cuff (A). Lymphocytic infiltrate was minimal, mainly organized in lymphocytic cuffs. CD3 positive lymphocytes (B), and scant CD20 positive lymphocytes (C). Positive immunostaining for TNFSF molecules TACI (D), TWEAK (E), Fn14 (F) and BAFF (G) was oserved in glial tumoral cells, vascular endothelium and inflammatory infiltrate. All pictures are taken under 40× magnification. (TIF) [file pone.0083250.s006.tif]

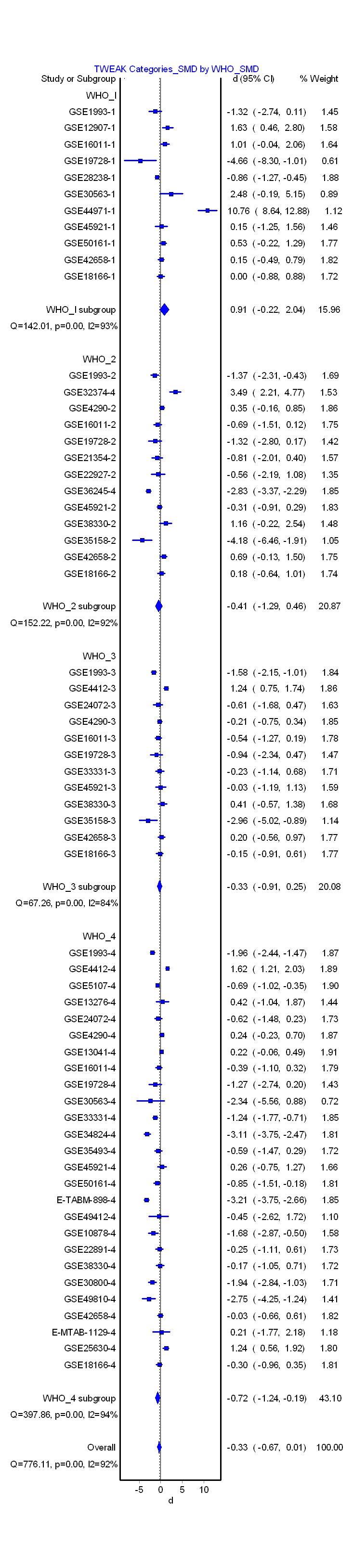

Supplement: Figure S7 — Meta-analysis of human gliomas' micro-arrays. Forest plot of TWEAK expression in 2083 tumor glioma specimens, stratified according to their WHO grade Results are presented as standardized mean differences (Cohen's d) between tumor and non-tumor samples. (TIF) [file pone.0083250.s007.tif]

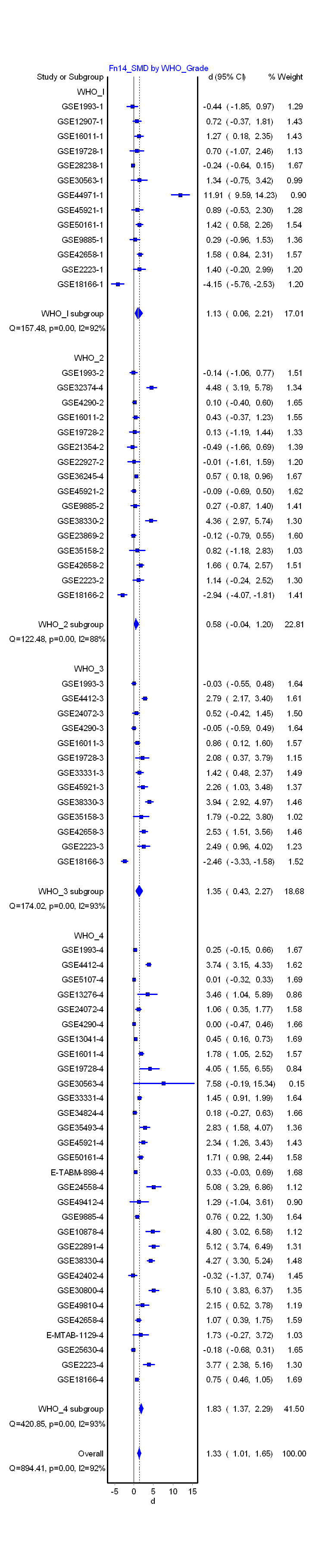

Supplement: Figure S8 — Meta-analysis of human gliomas' micro-arrays. Forest plot of Fn14 expression in 2083 tumor glioma specimens, stratified according to their WHO grade. Results are presented as standardized mean differences (Cohen's d) between tumor and non-tumor samples. (TIF) [file pone.0083250.s008.tif]
