# Supplementary material for: BAFF, APRIL, TWEAK, BCMA, TACI and Fn14 Proteins Are Related to Human Glioma Tumor Grade: Immunohistochemistry and Public Microarray Data Meta-Analysis
Source: PLoS One. 2013 Dec 20;8(12):e83250. doi: 10.1371/journal.pone.0083250 (PMC3869762; doi:10.1371/journal.pone.0083250)
Supplement: Table S1 — Identifiers of glioma micro-array studies used in the present work for the meta-analysis of the TNFSF members, including Supplementary References to these studies. (DOCX) [file pone.0083250.s009.docx]

**Table S1**

*Identifiers of glioma micro-array studies used in the present work for the meta-analysis of the TNFSF members*

| **Identifier GEO** | **Identifier Array Express** | **Ref** | **Total Cases** | **Control** | **Grade I** | **Grade II** | **Grade III** | **Grade IV** | **ProbeSet** | **ProbeSet Ref** | **Note** |
| --- | --- | --- | --- | --- | --- | --- | --- | --- | --- | --- | --- |
| GSE1993 | E-GEOD-1993 | [[1](#_ENREF_1)] | 65 | 0 | 2 | 5 | 19 | 39 | Affymetrix Human Genome U133A Array | GPL96 | It includes GSE2727 |
| GSE2223 |  | [[2](#_ENREF_2),[3](#_ENREF_3)] | 54 | 4 | 4 | 6 | 10 | 30 | SHFK | GPL1833 |  |
| GSE32374 | E-GEOD-32374 | [[4](#_ENREF_4)] | 21 | 0 | 0 | 0 | 0 | 21 | Affymetrix Human Genome U133 Plus 2.0 Array | GPL570 |  |
| GSE4290 | E-GEOD-4290 | [[5](#_ENREF_5)] | 180 | 23 | 0 | 45 | 31 | 81 | Affymetrix Human Genome U133 Plus 2.0 Array | GPL570 |  |
| GSE4412 | E-GEOD-4412 | [[6](#_ENREF_6)] | 85 | 0 | 0 | 0 | 26 | 59 | Affymetrix Human Genome U133A & U133B Arrays | GPL96, GPL97 |  |
| GSE5107 | E-GEOD--5107 | [[7](#_ENREF_7)] | 83 | 0 | 0 | 0 | 0 | 83 | Affymetrix Human Genome U133A Array | GPL96 |  |
| GSE9885 | E-GEOD-9885 | [[8](#_ENREF_8)] | 39 | 0 | 5 | 6 | 0 | 28 | Agilent-011521 Human 1A Microarray G4110A & G4110B | GPL885, GPL887 |  |
| GSE10878 |  | [[9](#_ENREF_9)] | 24 | 4 | 0 | 0 | 0 | 20 | Agilent-014850 Whole Human Genome Microarray | GPL6480 |  |
| GSE12907 | E-GEOD-12907 | [[10](#_ENREF_10)] | 25 | 4 | 21 | 0 | 0 | 0 | Affymetrix Human Genome U133A Array | GPL96 |  |
| GSE13041 | E-GEOD-13041 | [[11](#_ENREF_11)] | 267 | 0 | 0 | 0 | 0 | 267 | Affymetrix Human Genome U133A Array, Affymetrix Human Genome U133 Plus 2.0 Array, Affymetrix Human Genome U95 Version 2 Array | GPL96, GPL570, GPL8300 | It includes GSE4412 and GSE4271 |
| GSE13276 | E-GEOD-13276 | [[12](#_ENREF_12)] | 8 | 3 | 0 | 0 | 0 | 5 | Affymetrix Human Genome U133A Array | GPL96 | + 7 surrounding tissue |
| GSE16011 | E-GEOD-16011 | [[13](#_ENREF_13)] | 284 | 8 | 8 | 25 | 84 | 159 | Affymetrix Human Genome U133 Plus 2.0 Array | GPL570 |  |
| GSE18166 | E-GEOD-18166 | [[14](#_ENREF_14)] | 139 | 0 | 10 | 14 | 20 | 95 | DKFZ SC112 / human 9k BAC chip | GPL9189 |  |
| GSE19728 | E-GEOD-19728 | [[15](#_ENREF_15)] | 21 | 4 | 2 | 5 | 5 | 5 | Affymetrix Human Genome U133 Plus 2.0 | GPL570 |  |
| GSE21354 | E-GEOD-21354 |  | 14 | 4 | 0 | 10 | 0 | 0 | Affymetrix Human Genome U133 Plus 2.0 Array | GPL570 | Contains other tumors too |
| GSE22891 | E-GEOD-22891 | [[16](#_ENREF_16)] | 46 | 6 | 0 | 0 | 0 | 40 | Agilent-014850 Whole Human Genome Microarray | GPL4133 |  |
| GSE22927 | E-GEOD-22927 | [[17](#_ENREF_17)] | 8 | 2 | 0 | 6 | 0 | 0 | Affymetrix Human Genome U133 Plus 2.0 Array | GPL570 |  |
| GSE23869 | E-GEOD-23869 | [[18](#_ENREF_18)] | 10 | 0 | 0 | 10 | 0 | 0 | Illumina HumanHT-12 V3.0 beadchip | GPL6947 |  |
| GSE24072 | E-GEOD-24072 | [[19](#_ENREF_19)] | 32 | 0 | 0 | 0 | 7 | 25 | Affymetrix Human Genome U133A Array | GPL96 |  |
| GSE24558 | E-GEOD-24558 | [[20](#_ENREF_20)] | 11 | 0 | 0 | 0 | 0 | 11 | Affymetrix Human Exon 1.0 ST Array | GPL5175 |  |
| GSE25630 | E-GEOD-25630 | [[21-23](#_ENREF_21)] | 21 | 0 | 0 | 0 | 0 | 21 | Illumina HumanWG-6 v3.0 expression beadchip | GPL6884 |  |
| GSE28238 |  | [[24](#_ENREF_24)] | 40 | 0 | 40 | 0 | 0 | 0 | Affymetrix Human Genome U133 Plus 2.0 Array | GPL570 |  |
| GSE30563 | E-GEOD-30563 |  | 6 | 3 | 2 | 0 | 0 | 1 | Affymetrix Human Genome U133 Plus 2.0 Array | GPL570 |  |
| GSE30800 | E-GEOD-30800 |  | 21 | 0 | 0 | 0 | 0 | 21 | Agilent-021529 Human CGH Whole Genome Microarray | GPL8737 |  |
| GSE33331 | E-GEOD-33331 | [[25](#_ENREF_25)] | 26 | 0 | 0 | 0 | 5 | 21 | Affymetrix Human Genome U133 Plus 2.0 Array | GPL570 |  |
| GSE34824 | E-GEOD-34824 | [[26](#_ENREF_26)] | 27 | 0 | 0 | 0 | 0 | 27 | Affymetrix Human Genome U133 Plus 2.0 Array | GPL570 |  |
| GSE35158 | E-GEOD-35158 | [[27](#_ENREF_27)] | 81 | 1 | 0 | 29 | 51 | 0 | Illumina HumanHT-12 WG-DASL V4.0 R2 expression beadchip | GPL14951 |  |
| GSE35493 | E-GEOD-35493 | [[28](#_ENREF_28)] | 21 | 9 | 0 | 0 | 0 | 12 | Affymetrix Human Genome U133 Plus 2.0 Array | GPL570 | Contains other tumors too |
| GSE36245 | E-GEOD-36245 | [[29](#_ENREF_29)] | 46 | 0 | 0 | 0 | 0 | 46 | Affymetrix Human Genome U133 Plus 2.0 Array | GPL570 |  |
| GSE38330 |  | [[30](#_ENREF_30)] | 20 | 0 | 0 | 3 | 7 | 10 | Agilent-014850 Whole Human Genome Microarray, Agilent-020087 human whole genome | GPL4133, GPL15287 |  |
| GSE42402 | E-GEOD-42402 | [[31](#_ENREF_31)] | 7 | 0 | 0 | 0 | 0 | 7 | Agilent-014850 Whole Human Genome Microarray | GPL4133 |  |
| GSE42658 | E-GEOD-42658 |  | 73 | 16 | 19 | 9 | 10 | 19 | Illumina HumanHT-12 V3.0 expression beadchip | GPL6947 |  |
| GSE44971 | E-GEOD-44971 | [[32](#_ENREF_32)] | 58 | 9 | 49 | 0 | 0 | 0 | Affymetrix Human Genome U133 Plus 2.0 Array | GPL570 |  |
| GSE45921 | E-GEOD-45921 |  | 22 | 0 | 2 | 13 | 3 | 4 | Affymetrix Human Genome U133 Plus 2.0 Array | GPL570 |  |
| GSE49412 | E-GEOD-49412 |  | 6 | 1 | 0 | 0 | 0 | 5 | Affymetrix Human Gene 1.0 ST Array | GPL15648 |  |
| GSE49810 | E-GEOD-49810 |  | 5 | 0 | 0 | 0 | 0 | 5 | Agilent-014693 Human Genome CGH Microarray 244A (Probe name version) | GPL9128 |  |
| GSE50161 | E-GEOD-50161 |  | 62 | 13 | 15 | 0 | 0 | 34 | Affymetrix Human Genome U133 Plus 2.0 Array | GPL570 | Contains other tumors too |
|  | E-MTAB-1129 |  | 69 | 1 | 0 | 0 | 0 | 68 | [Illumina HumanHT-12_V4_0_R1_15002873_B](http://www.ebi.ac.uk/arrayexpress/arrays/A-MEXP-2210/?ref=E-MTAB-1129) | A-MEXP-2210 |  |
|  | E-TABM-898 | [[33](#_ENREF_33)] | 56 | 0 | 0 | 0 | 0 | 56 | Affymetrix Human Genome U133 Plus 2.0 Array | GPL570 |  |
| **Totals** | **39** |  | **2083** | **115** | **179** | **186** | **278** | **1325** |  |  |  |

**Supplemental References**

1. Petalidis LP, Oulas A, Backlund M, Wayland MT, Liu L, et al. (2008) Improved grading and survival prediction of human astrocytic brain tumors by artificial neural network analysis of gene expression microarray data. Mol Cancer Ther 7: 1013-1024.

2. Bredel M, Bredel C, Juric D, Duran GE, Yu RX, et al. (2006) Tumor necrosis factor-alpha-induced protein 3 as a putative regulator of nuclear factor-kappaB-mediated resistance to O6-alkylating agents in human glioblastomas. J Clin Oncol 24: 274-287.

3. Bredel M, Bredel C, Juric D, Harsh GR, Vogel H, et al. (2005) Functional network analysis reveals extended gliomagenesis pathway maps and three novel MYC-interacting genes in human gliomas. Cancer Res 65: 8679-8689.

4. Macy ME, Birks DK, Barton VN, Chan MH, Donson AM, et al. (2012) Clinical and molecular characteristics of congenital glioblastoma. Neuro Oncol 14: 931-941.

5. Sun L, Hui AM, Su Q, Vortmeyer A, Kotliarov Y, et al. (2006) Neuronal and glioma-derived stem cell factor induces angiogenesis within the brain. Cancer Cell 9: 287-300.

6. Freije WA, Castro-Vargas FE, Fang Z, Horvath S, Cloughesy T, et al. (2004) Gene expression profiling of gliomas strongly predicts survival. Cancer Res 64: 6503-6510.

7. Tso CL, Shintaku P, Chen J, Liu Q, Liu J, et al. (2006) Primary glioblastomas express mesenchymal stem-like properties. Mol Cancer Res 4: 607-619.

8. Marucci G, Morandi L, Magrini E, Farnedi A, Franceschi E, et al. (2008) Gene expression profiling in glioblastoma and immunohistochemical evaluation of IGFBP-2 and CDC20. Virchows Arch 453: 599-609.

9. de Tayrac M, Etcheverry A, Aubry M, Saikali S, Hamlat A, et al. (2009) Integrative genome-wide analysis reveals a robust genomic glioblastoma signature associated with copy number driving changes in gene expression. Genes Chromosomes Cancer 48: 55-68.

10. Wong KK, Chang YM, Tsang YT, Perlaky L, Su J, et al. (2005) Expression analysis of juvenile pilocytic astrocytomas by oligonucleotide microarray reveals two potential subgroups. Cancer Res 65: 76-84.

11. Lee Y, Scheck AC, Cloughesy TF, Lai A, Dong J, et al. (2008) Gene expression analysis of glioblastomas identifies the major molecular basis for the prognostic benefit of younger age. BMC Med Genomics 1: 52.

12. Mangiola A, Saulnier N, De Bonis P, Orteschi D, Sica G, et al. (2013) Gene expression profile of glioblastoma peritumoral tissue: an ex vivo study. PLoS One 8: e57145.

13. Gravendeel LA, Kouwenhoven MC, Gevaert O, de Rooi JJ, Stubbs AP, et al. (2009) Intrinsic gene expression profiles of gliomas are a better predictor of survival than histology. Cancer Res 69: 9065-9072.

14. Toedt G, Barbus S, Wolter M, Felsberg J, Tews B, et al. (2011) Molecular signatures classify astrocytic gliomas by IDH1 mutation status. Int J Cancer 128: 1095-1103.

15. Liu Z, Xie M, Yao Z, Niu Y, Bu Y, et al. (2013) Three meta-analyses define a set of commonly overexpressed genes from microarray datasets on astrocytomas. Mol Neurobiol 47: 325-336.

16. Etcheverry A, Aubry M, de Tayrac M, Vauleon E, Boniface R, et al. (2010) DNA methylation in glioblastoma: impact on gene expression and clinical outcome. BMC Genomics 11: 701.

17. Chow LM, Endersby R, Zhu X, Rankin S, Qu C, et al. (2011) Cooperativity within and among Pten, p53, and Rb pathways induces high-grade astrocytoma in adult brain. Cancer Cell 19: 305-316.

18. Jones DT, Mulholland SA, Pearson DM, Malley DS, Openshaw SW, et al. (2011) Adult grade II diffuse astrocytomas are genetically distinct from and more aggressive than their paediatric counterparts. Acta Neuropathol 121: 753-761.

19. Garcia JL, Couceiro J, Gomez-Moreta JA, Gonzalez Valero JM, Briz AS, et al. (2012) Expression of VAV1 in the tumour microenvironment of glioblastoma multiforme. J Neurooncol 110: 69-77.

20. Wang R, Chadalavada K, Wilshire J, Kowalik U, Hovinga KE, et al. (2010) Glioblastoma stem-like cells give rise to tumour endothelium. Nature 468: 829-833.

21. Wang Y, Li S, Chen L, You G, Bao Z, et al. (2012) Glioblastoma with an oligodendroglioma component: distinct clinical behavior, genetic alterations, and outcome. Neuro Oncol 14: 518-525.

22. Yan W, Zhang W, You G, Zhang J, Han L, et al. (2012) Molecular classification of gliomas based on whole genome gene expression: a systematic report of 225 samples from the Chinese Glioma Cooperative Group. Neuro Oncol 14: 1432-1440.

23. Chen L, Zhang W, Yan W, Han L, Zhang K, et al. (2012) The putative tumor suppressor miR-524-5p directly targets Jagged-1 and Hes-1 in glioma. Carcinogenesis 33: 2276-2282.

24. Mascelli S, Barla A, Raso A, Mosci S, Nozza P, et al. (2013) Molecular fingerprinting reflects different histotypes and brain region in low grade gliomas. BMC Cancer 13: 387.

25. Donson AM, Birks DK, Schittone SA, Kleinschmidt-DeMasters BK, Sun DY, et al. (2012) Increased immune gene expression and immune cell infiltration in high-grade astrocytoma distinguish long-term from short-term survivors. J Immunol 189: 1920-1927.

26. Schwartzentruber J, Korshunov A, Liu XY, Jones DT, Pfaff E, et al. (2012) Driver mutations in histone H3.3 and chromatin remodelling genes in paediatric glioblastoma. Nature 482: 226-231.

27. Gorovets D, Kannan K, Shen R, Kastenhuber ER, Islamdoust N, et al. (2012) IDH mutation and neuroglial developmental features define clinically distinct subclasses of lower grade diffuse astrocytic glioma. Clin Cancer Res 18: 2490-2501.

28. Birks DK, Donson AM, Patel PR, Sufit A, Algar EM, et al. (2013) Pediatric rhabdoid tumors of kidney and brain show many differences in gene expression but share dysregulation of cell cycle and epigenetic effector genes. Pediatr Blood Cancer 60: 1095-1102.

29. Sturm D, Witt H, Hovestadt V, Khuong-Quang DA, Jones DT, et al. (2012) Hotspot mutations in H3F3A and IDH1 define distinct epigenetic and biological subgroups of glioblastoma. Cancer Cell 22: 425-437.

30. Engler JR, Robinson AE, Smirnov I, Hodgson JG, Berger MS, et al. (2012) Increased microglia/macrophage gene expression in a subset of adult and pediatric astrocytomas. PLoS One 7: e43339.

31. Parker BC, Annala MJ, Cogdell DE, Granberg KJ, Sun Y, et al. (2013) The tumorigenic FGFR3-TACC3 gene fusion escapes miR-99a regulation in glioblastoma. J Clin Invest 123: 855-865.

32. Lambert SR, Witt H, Hovestadt V, Zucknick M, Kool M, et al. (2013) Differential expression and methylation of brain developmental genes define location-specific subsets of pilocytic astrocytoma. Acta Neuropathol 126: 291-301.

33. Ducray F, de Reynies A, Chinot O, Idbaih A, Figarella-Branger D, et al. (2010) An ANOCEF genomic and transcriptomic microarray study of the response to radiotherapy or to alkylating first-line chemotherapy in glioblastoma patients. Mol Cancer 9: 234.
