# Supplementary material for: BAFF, APRIL, TWEAK, BCMA, TACI and Fn14 Proteins Are Related to Human Glioma Tumor Grade: Immunohistochemistry and Public Microarray Data Meta-Analysis
Source: PLoS One. 2013 Dec 20;8(12):e83250. doi: 10.1371/journal.pone.0083250 (PMC3869762; doi:10.1371/journal.pone.0083250)
Supplement: Table S2 — Primary antibodies and detection kits used in the study. (DOCX) [file pone.0083250.s010.docx]

**Table S2**

*Primary antibodies and detection kits used in the study*

MW=microwaves pre-treatment of slides for antigen retrieval.

| **Antibody** | **Code** | **Company** | **Pretreatment** | **Dilution** | **Incubation Time** | **Detection Kit** |
| --- | --- | --- | --- | --- | --- | --- |
| **Primary Antibodies** | | | | | | |
| APRIL | ALX-804-149-C100, Mouse monoclonal | ENZO^1^ | MW | 1/100 | 1h | AP polymer |
| BAFF | ALX-804-131-C100, Rat monoclonal | ENZO^1^ | MW | 1/200 | 1h | AP polymer |
| BAFF-R | AF1162, Goat polyclonal | R&D^2^ | MW | 1/100 | 1h | K0689 |
| BCMA | ALX- 804-151-C100, Rat monoclonal | ENZO^1^ | MW | 1/100 | 30' | K1500 |
| BCMA-FITC | NBP1-97655, Rat monoclonal | Novus^3^ | MW | 1/100 | 1h | - |
| TACI | Sc-80335, Mouse monoclonal | Santa Cruz^4^ | MW | 1/150 | 1h | AP polymer |
| TWEAK | Sc-12405, Goat polyclonal | Santa Cruz^4^ | EDTA | 1/100 | 30' | K1500 |
| Fn14 | Sc-56250, Mouse monoclonal | Santa Cruz^4^ | EDTA | 1/100 | 30' | K1500 |
| CD68 | MS-397-P0, Mouse Monoclonal | Thermo^5^ | Proteinase K | 1/200 | 45’ | Quanto polymer |
| CD3 | A 0452, Rabbit polyclonal | DAKO^6^ | MW | 1/500 | 60’ | Quanto polymer |
| CD20 | M0755, Mouse Monoclonal | DAKO^6^ | EDTA | 1/500 | 60’ | Quanto polymer |
| **Detection Kits** | | | | | | |
| Quanto polymer | TL-060-QHD, Thermo | Thermo^5^ | UltraVision Quanto Detection System, DAB | | | |
| AP polymer | TL-125-AL, Lab Vision | Thermo^5^ | Ultra vision detection system Ap Polymer, Fast Red | | | |
|  | | | | | | |
|  | | | | | | |
| K1500 | K1500 | DAKO^6^ | Catalyzed signal amplification system, DAB | | | |
|  | | | | | | |
| K0689 | K0689 | DAKO^6^ | Lsab system Ap, Fast Red | | | |

^1^ ENZO Life Sciences, Farmingdale, New York, NY, ^2^ R&D Systems, Minneapolis, MN, USA, ^3^ Novus Biologicals, Cambridge, UK. ^4^ Santa Cruz Biotechnology, CA, USA, ^5^ Thermo Fisher Scientific, Fremont, CA, ^6^ Dako, Glostrup, Denmark
