# Supplementary material for: BAFF, APRIL, TWEAK, BCMA, TACI and Fn14 Proteins Are Related to Human Glioma Tumor Grade: Immunohistochemistry and Public Microarray Data Meta-Analysis
Source: PLoS One. 2013 Dec 20;8(12):e83250. doi: 10.1371/journal.pone.0083250 (PMC3869762; doi:10.1371/journal.pone.0083250)
Supplement: Table S3 — Non-parametric correlation of standardized mean differences of micro-array studies. (DOCX) [file pone.0083250.s011.docx]

**Table S3**

*Non-parametric correlation of standardized mean differences of micro-array studies.*

|  | | APRIL | BAFF | BAFFR | TACI | BCMA | TWEAK | Fn14 |
| --- | --- | --- | --- | --- | --- | --- | --- | --- |
| APRIL | Correlation Coefficient | 1.000 | **.402^*^** | .075 | .230 | .023 | **.576^**^** | **.333^**^** |
|  | Sig. (2-tailed) |  | .012 | .650 | .098 | .864 | .000 | **.010** |
|  | N | 59 | 38 | 39 | 53 | 57 | 52 | **59** |
| BAFF | Correlation Coefficient |  | 1.000 | .251 | -.229 | .174 | .180 | **.524^**^** |
|  | Sig. (2-tailed) |  |  | .104 | .144 | .265 | .249 | **.000** |
|  | N |  |  | 43 | 42 | 43 | 43 | **44** |
| BAFFR | Correlation Coefficient |  |  | 1.000 | .140 | .031 | .081 | -.211 |
|  | Sig. (2-tailed) |  |  |  | .342 | .835 | .586 | .146 |
|  | N |  |  |  | 48 | 48 | 48 | 49 |
| TACI | Correlation Coefficient |  |  |  | 1.000 | **.303^*^** | -.035 | -.046 |
|  | Sig. (2-tailed) |  |  |  |  | **.015** | .792 | .714 |
|  | N |  |  |  |  | **64** | 60 | 65 |
| BCMA | Correlation Coefficient |  |  |  |  | 1.000 | -.117 | **.268^*^** |
|  | Sig. (2-tailed) |  |  |  |  |  | .372 | **.026** |
|  | N |  |  |  |  |  | 60 | **69** |
| TWEAK | Correlation Coefficient |  |  |  |  |  | 1.000 | .191 |
|  | Sig. (2-tailed) |  |  |  |  |  |  | .136 |
|  | N |  |  |  |  |  |  | 62 |
