# Supplementary material for: BAFF, APRIL, TWEAK, BCMA, TACI and Fn14 Proteins Are Related to Human Glioma Tumor Grade: Immunohistochemistry and Public Microarray Data Meta-Analysis
Source: PLoS One. 2013 Dec 20;8(12):e83250. doi: 10.1371/journal.pone.0083250 (PMC3869762; doi:10.1371/journal.pone.0083250)
Supplement: Table S4 — H-score of the TNFSF members in gliomas. (DOCX) [file pone.0083250.s012.docx]

**Table S4**

*H-index of individual IHC cases analyzed in the present study*

| **Ref#** | **Grade** | **TWEAK** | **Fn14** | **APRIL** | **BAFF** | **BAFFR** | **BCMA** | **TACI** |
| --- | --- | --- | --- | --- | --- | --- | --- | --- |
| 59981 | 1 |  | 255 | 44 | 214 | 0 | 290 | 171 |
| 64094 | 1 | 233 | 270 | 145 | 178 | 0 | 293 | 287 |
| 108237 | 1 |  | 215 | 99 | 265 | 0 |  | 282 |
| 21773 | 2 |  | 247 | 199 | 250 | 0 | 265 | 287 |
| 44008 | 2 | 291 | 175 | 124 | 204 | 0 | 260 |  |
| 44751 | 2 | 251 | 290 | 17 | 181 | 0 | 290 | 190 |
| 45903 | 2 | 197 | 165 | 200 | 84 | 0 | 139 | 5 |
| 47047 | 2 | 122 | 290 | 30 | 161 | 0 | 191 | 232 |
| 72132 | 2 | 170 | 228 | 41 | 208 | 0 | 160 | 25 |
| 75405 | 2 | 258 | 200 | 122 | 105 | 0 | 296 | 186 |
| 78715 | 2 | 247 | 220 | 39 | 176 | 0 | 192 | 147 |
| 102291 | 2 | 288 | 280 | 74 | 186 | 0 | 160 | 270 |
| 579/09 | 2 | 292 | 275 | 24 | 24 | 0 | 207 | 251 |
| 23717 | 3 |  | 230 | 155 | 99 | 0 | 205 | 192 |
| 48483 | 3 |  | 300 | 274 | 144 | 0 | 295 | 283 |
| 53740 | 3 | 279 | 295 | 257 | 5 | 0 | 272 | 236 |
| 72299 | 3 | 294 | 300 | 120 | 185 | 0 | 258 | 285 |
| 88057 | 3 |  | 210 | 180 | 116 | 0 |  |  |
| 89070 | 3 | 284 | 148.9 | 280 | 222 | 0 | 277 | 272 |
| 97102 | 3 | 225 | 210 | 250 | 280 | 0 | 286 | 289 |
| 98102 | 3 |  | 265 | 66 | 98 | 0 | 277 |  |
| 97535 | 3 |  | 213 | 274 | 178 | 0 | 214 | 240 |
| 132872 | 3 | 287 | 274 | 2 | 114 | 0 | 263 | 278 |
| 21950 | 4 | 274 | 195 | 103 | 203 | 0 | 252 |  |
| 22505 | 4 | 185 | 250 | 295 | 140 | 0 | 286 | 264 |
| 26033 | 4 | 262 | 205 | 274 | 119 | 0 | 287 | 263 |
| 29056 | 4 |  | 190 | 250 | 204 | 0 | 282 |  |
| 31013 | 4 | 247 | 230 | 28 |  | 0 | 227 | 242 |
| 32890 | 4 | 212 | 260 | 16 |  | 0 | 294 | 212 |
| 37149 | 4 | 251 | 245 | 169 | 270 | 0 | 264 |  |
| 37237 | 4 |  | 204 | 44 | 134 | 0 |  |  |
| 39279 | 4 | 237 | 295 | 31 | 171 | 0 | 291 |  |
| 44152 | 4 |  | 125 | 200 | 200 | 0 | 270 | 291 |
| 45788 | 4 | 283 | 275 | 56 | 0 | 0 | 291 | 131 |
| 46398 | 4 | 283 | 230 | 295 | 134 | 0 | 265 | 193 |
| 48211 | 4 |  | 245 | 77 | 77 | 0 | 255 | 262 |
| 51481 | 4 | 278 | 270 | 280 | 115 | 0 | 279 | 269 |
| 51696 | 4 | 250 | 160 | 163 | 153 | 0 | 203 | 279 |
| 53256 | 4 |  | 152 | 281 | 149 | 0 | 285 |  |
| 54729 | 4 |  |  | 269 | 185 | 0 | 292 | 293 |
| 55270 | 4 |  | 176 | 253 | 176 | 0 | 281 |  |
| 60164 | 4 |  | 230 | 170 | 190 | 0 | 218 |  |
| 64520 | 4 |  | 175 | 56 | 212 | 0 | 242 |  |
| 71695 | 4 |  | 175 | 126 | 280 | 0 | 280 | 212 |
| 72048 | 4 | 286 | 260 | 240 | 139 | 0 | 246 | 198 |
| 86928 | 4 | 254 | 240 | 234 | 105 | 0 | 202 | 237 |
| 87521 | 4 | 261 | 285 | 94 | 45 | 0 | 289 |  |
| 107535 | 4 | 223 | 200 | 140 | 115 | 0 | 184 | 129 |
| 108572 | 4 |  |  | 177 | 240 | 0 | 229 | 239 |
| 110376 | 4 | 178 | 275 | 8 | 147 | 0 | 262 | 257 |
| 122864 | 4 |  | 174 | 131 |  | 0 | 280 | 244 |
| 129885 | 4 | 244 | 276 | 70 | 98 | 0 | 184 | 214 |
| 130429 | 4 | 132 | 36 | 288 |  | 0 | 108 | 215 |
| 660/09 | 4 | 262 | 245 | 77 | 3 | 0 |  | 286 |
| 828/09 | 4 | 31 | 247 | 9 |  | 0 |  |  |
| 4665/09 | 4 | 221 | 43 | 162 |  | 0 | 263 | 221 |
