# Supplementary material for: BAFF, APRIL, TWEAK, BCMA, TACI and Fn14 Proteins Are Related to Human Glioma Tumor Grade: Immunohistochemistry and Public Microarray Data Meta-Analysis
Source: PLoS One. 2013 Dec 20;8(12):e83250. doi: 10.1371/journal.pone.0083250 (PMC3869762; doi:10.1371/journal.pone.0083250)
Supplement: Flowchart S1 — PRISMA Flowchart explaining the selection of studies included in the meta-analysis presented here. (DOC) [file pone.0083250.s014.doc]

**Identification**

**Screening**

**Eligibility**

**Included**

# of records identified through database searching: 423 Array Express

# of additional records identified through other sources: 388 GEO

# of records after duplicates removed: 445

# of records screened: 445

# of records excluded: 406

# of data sets assessed for eligibility: 39

# of studies included in qualitative synthesis: 39

# of data sets excluded, with reasons: 0

# of studies included in quantitative synthesis (meta-analysis): 39
